# Data quality challenges of AIGC application in smart agriculture

**DOI:** 10.3389/frai.2025.1640805

**Published:** 2025-09-23

**Authors:** Yingxue Ren, Yitong Qu, Runzeng Gao

**Affiliations:** ^1^Business School, Nankai University, Tianjin, China; ^2^School of Economics and Management, Tiangong University, Tianjin, China

**Keywords:** smart agriculture, data quality, data noise, data fog, data islands, AIGC

## Abstract

In recent years, China’s agricultural development has gradually shifted from digital agriculture to smart agriculture. At the same time, with the participation of AIGC, the decision-making system of smart agriculture is also facing numerous data challenges. In this study, we employed a comprehensive quality improvement approach to ad-dress these challenges. The methodology involves three phases: (1) Detection and removal of data noise through advanced cleaning techniques and preprocessing methods; (2) Unified data standards and formats to ensure seamless integration across di-verse data sources; and (3) Strengthening agricultural infrastructure to prevent data islands and promote equitable data distribution. Our analysis reveals that data noise significantly impacts precision agriculture, leading to biased decisions and resource wastage. Data fog, resulting from heterogeneous data sources and weak inter-source correlations, complicates decision-making processes. Additionally, data islands hinder data sharing and integration, exacerbated by uneven data development across regions. Systematic implementation of standardized quality control protocols is essential for enhancing smart agricultural systems and ensuring sustainable development. This study offers a novel perspective on enhancing data quality in AIGC-driven smart agriculture by integrating the Juran quality improvement model.

## 1 Introduction

In October 2024, the Ministry of Agriculture and Rural Affairs issued the “National Smart Agriculture Action Plan (2024–2028)” (MARD, 2024), shifting China’s agricultural development from digital agriculture to smart agriculture. By generating text, images, audio, and video content, Artificial Intelligence Generated Content (AIGC), combined with advanced technologies like natural language processing, computer vision, and machine learning (Waleed Khalid et al., 2024), can offer precision agriculture decision (Bongiovanni and Lowenberg-Deboer, 2004) and verify compliance with good agricultural practices (GAP) criteria (De Baerdemaeker, 2013), thereby enhancing agricultural production efficiency and sustainability (Ilcic et al., 2025).

However, AIGC also introduces complexities in data quality, posing challenges for smart agriculture. To start with, problems such as algorithmic bias, unstable data sources, and lack of model transparency can lead to data bias (Dehghani et al., 2024), creating data noise (Martín et al., 2024). For instance, biased training data may result in misleading predictions about crop growth or pest and disease outbreaks (Jabed and Azmi Murad, 2024). Furthermore, AIGC exacerbates data fog, as integrating and interpreting data from diverse sources and formats becomes complex. This complexity hinders agricultural producers’ ability to effectively use data for decision-making (Ribeiro Junior et al., 2022). Additionally, AIGC can intensify data islands, creating barriers to data sharing and integration between systems and departments, and impeding data flow and analysis (Jakku et al., 2019). For example, a smart irrigation system may lack access to soil moisture data from the environmental system, reducing irrigation efficiency (Morchid et al., 2024). While the detrimental effects of data noise, fog, and islands on smart agriculture are increasingly recognized, there remains a critical gap in systematically addressing these intertwined data quality challenges through a unified, process-oriented quality improvement framework within the specific context of AIGC adoption.

**Figure 1:**
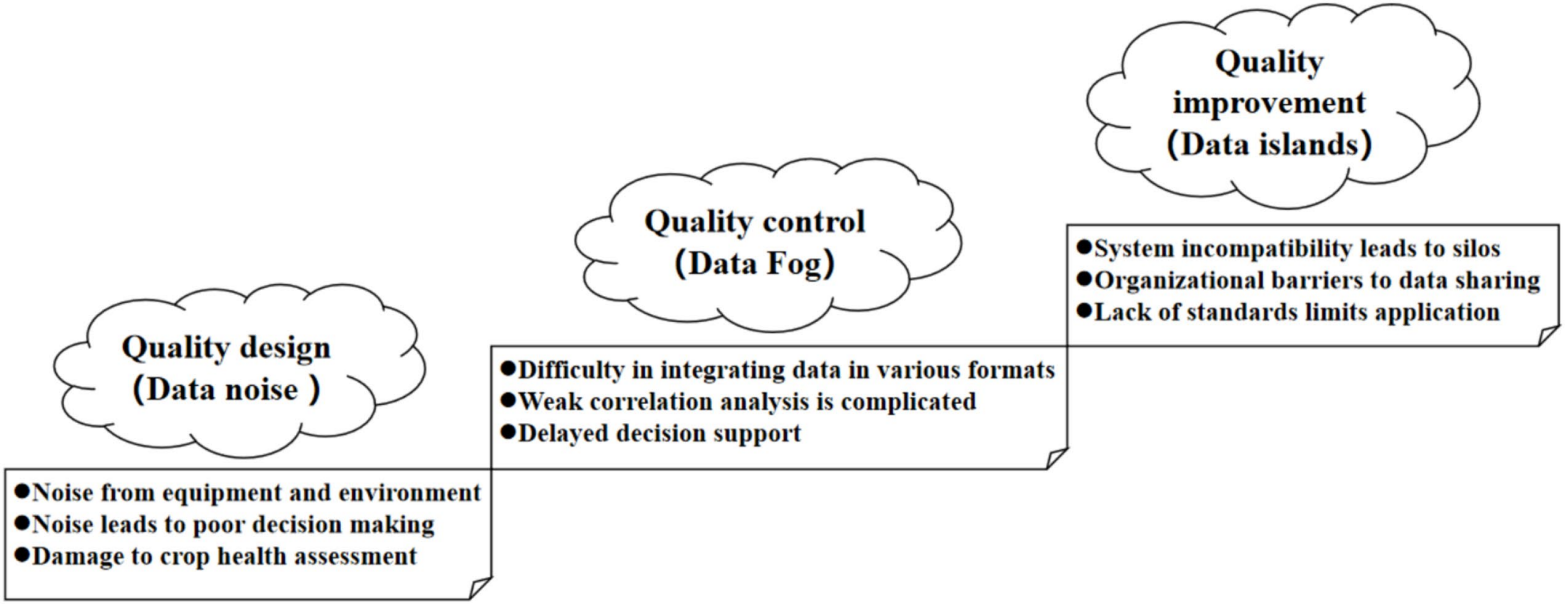
AIGC generated data quality improvement roadmap in Smart Agriculture.

Addressing these challenges is crucial for AIGC application in smart agriculture. Quality loops, a conceptual model emphasizing continuous improvement from a quality perspective (Tesfay, 2021), was proposed as a lens to gain insights on data quality challenges in the AIGC application of smart agriculture (See Figure 1). This study explicitly focuses on analyzing and proposing solutions for three core data quality challenges hindering AIGC-driven smart agriculture: data noise (affecting accuracy and reliability), data fog (hindering integration and interpretation), and data islands (impeding sharing and flow). With the lens, this viewpoint analyzes the root causes of these challenges, explains the potential issues from the perspective of smart agriculture, and demonstrates their impact in the long term. By deeply exploring data quality challenges in smart agriculture, this viewpoint demonstrates typical data quality challenges in AIGC applications in the view of smart agriculture. Insights could be valuable for researchers, and practitioners, and inform future technology applications.

The remainder of this paper is organized as follows. Section 2 analyzes data noise within the quality design phase. Section 3 examines data fog in the quality control phase. Section 4 discusses data islands in the quality improvement phase. Section 5 synthesizes the findings and provides targeted suggestions for mitigating these challenges. Finally, Section 6 concludes the paper.

## 2 Data noise in the quality design phase

Noise is an unavoidable problem, which affects the data collection and data preparation processes in Data Mining applications, where errors commonly occur (García et al., 2015). Data noise, which encompasses errors and interference within datasets, poses a significant challenge in the domain of smart agriculture, particularly with the integration of AIGC (Martín et al., 2024). Sensor faults, including those due to equipment limitations and wear from extended use, can introduce errors during data acquisition (Li et al., 2020). Additionally, environmental fluctuations such as temperature, humidity, and wind, can impact sensor readings and amplify data noise (Cai et al., 2018). Data transmission from acquisition to storage points may also be compromised by network constraints and signal degradation, leading to data corruption or loss (Brinkmann et al., 2009). Human errors during data entry and processing, especially in manual operations (Paul and Lars, 2003), are also significant sources of data noise and are inherently challenging to eliminate.

The presence of data noise is a common problem that produces several negative consequences in smart agriculture. It can result in biased agricultural decisions, particularly within precision agriculture technologies (Tey and Brindal, 2012), such as irrigation, fertilization, and pest management. Biased agricultural decisions may lead to resource wastage, including excessive water use and pesticide application, which hinder agriculture sustainability (Bongiovanni and Lowenberg-Deboer, 2004). Moreover, data noise can impair the precise assessment of compliance with GAP, which impacts the quality of agricultural production. It can also lead to increased maintenance and calibration expenses, as well as financial losses due to flawed decision-making. Consequently, data noise is a critical issue in smart agriculture, influenced by multiple factors and exerting a broad impact on agricultural operations.

A high-quality dataset is one that accurately represents real-world phenomena, is comprehensive, and is free from biases (Gong et al., 2023). In the AIGC context, addressing data noise has become an essential component of smart agriculture. The performance of smart agriculture will heavily depend on the quality of the dataset, but also on the robustness against the noise. To enhance the efficiency and effectiveness of smart agriculture, a thorough diagnostic of the sources of data noise and the implementation of cleaning methods are imperative (Xiong et al., 2006). This necessitates comprehensive consideration of data quality control and optimization during the design, deployment, and upkeep of AIGC technology, ensuring that smart agriculture systems can built on accurate and dependable data, thereby facilitating precision agriculture.

## 3 Data fog in the quality control phase

Data fog is caused by the complexity of heterogeneous data from multiple sources and the ambiguity of the relationship between data (Kumari et al., 2019). In the context of AIGC technology, this issue has become even more prominent. While AIGC technology offers volumes of data with a wide variety that can be captured, analyzed, and used for decision-making, it also adds the heterogeneity and complexity of data. In smart agriculture, data from different sources, such as sensors and robots (Wolfert et al., 2017), can become misguided in data fog if not effectively integrated, affecting the accuracy of information and the timeliness of decisions.

Data fog arises from several key issues. Data in smart agriculture comes from a variety of devices of various stakeholders. These data sets often have different formats and standards, making their integration and analysis complicated (Cheng et al., 2024). Second, the correlation between different data sources is quite weak, and the lack of standardized protocols to correlate these datasets exacerbates the difficulty of integration (Bimonte et al., 2024). Finally, existing data processing technologies may not be sufficient to handle large, multi-source, heterogeneous data, thus limiting the utilization of data (Hazra et al., 2023). The quality and usefulness of data integration depend on the existence and adoption of standards, shared formats, and mechanisms (Lapatas et al., 2015). These problems not only increase the complexity of data processing but also hinder the application of data in systematic decision-making, affecting the precision and accuracy of agricultural production.

The impact of data fog on smart farming is multifaceted. Firstly, the diverse formats and standards of agricultural data make integration and analysis difficult (Leonelli et al., 2017), preventing stakeholders from extracting valuable and intime information. Secondly, data fog increases the difficulty of data processing, reduces efficiency, delays the time for decision-makers to obtain accurate data support, and affects the level of intelligence and precision of agricultural production. In addition, data fog can lead to unsustainable production, as growers may fail to adjust their agricultural practices based on real-time data (Wolfert et al., 2017), thus failing to achieve the goals of precision agriculture. Therefore, solving the problem of data fog and enhancing data integration and analysis capabilities are crucial to smart agriculture.

## 4 Data islands in the quality improvement phase

Data islands, a critical issue in smart agriculture, denote the inability to connect and share data across disparate systems or departments due to system incompatibilities, organizational barriers, and the absence of uniform standards (Philipp and David, 2020; Radauer et al., 2023). These impediments to information flow not only obstruct data integration and analysis but also precipitate decision-making errors and resource wastage. For instance, the isolation of agricultural enterprises’ sales and production data, resulting in a lack of understanding of market demand by the production department, leads to mismatched planting varieties and quantities, substantial economic loss, and even food waste.

The main cause of data islands is the uneven development of agricultural data. When data scarcity occurs, it weakens the connections and further exacerbates data fragmentation (Jones et al., 2017), ultimately leading to the formation of data islands. This disparity in data development can exacerbate economic inequalities among different regions (Wang, 2015), as farmers in less developed areas may lack access to the advanced technologies and insights available to those in more data-rich regions.

Without comprehensive data, it becomes challenging to make informed decisions about systematic problems, such as pesticide application (Pan et al., 2021). This can lead to inefficient use of resources, increased costs, and potentially lower crop yields and quality. This isolation of data island not only restricts the effectiveness of individual farming operations but also hinders the overall performance of smart agriculture on a broader scale, causing the shortage of a barrel.

## 5 Discussion

The analysis presented in the preceding sections underscores the profound impact of data noise, fog, and islands on the efficacy of AIGC-driven smart agriculture. These challenges are not isolated: data noise can obscure signals within individual datasets, complicating integration (fog) and rendering shared data less reliable (exacerbating island effects) (Anand et al., 2024). Data fog hinders the correlation of information necessary to overcome silos (islands). Conversely, data islands prevent access to diverse data sources needed to contextualize and clean noisy data or resolve fog ambiguities (Mishra et al., 2023). While existing research often tackles these issues individually, the quality loop perspective adopted here reveals their interconnected nature and the necessity for a holistic, phase-specific approach spanning the entire data lifecycle—from design and acquisition (noise), through integration and processing (fog), to sharing and utilization (islands). Successfully mitigating these intertwined challenges is paramount for realizing the full potential of AIGC in enabling truly precise, efficient, and sustainable smart agricultural systems (Martín et al., 2024).

## 6 Suggestion

The application of AIGC technology brings unprecedented changes to agricultural production, enabling more intelligent and data-driven decision-making processes. However, challenges such as data noise, data fog, and data islands have gained increasing attention from researchers, as they significantly affect the effectiveness of AIGC implementations. For instance, studies by Gupta and Gupta (2019) have highlighted the detrimental effects of data noise on prediction accuracy, while Sadri et al. (2021) discussed the complexities introduced by data fog in multi-source data environments. Additionally, Sullivan et al. (2024) emphasized the barriers posed by data islands to data sharing and collaborative agricultural management. This viewpoint offers suggestions from a unique quality improvement perspective to analyze and mitigate these challenges, providing a structured approach to enhance data reliability and usability in smart agriculture.

To start with, in the “quality design” phase of the quality loop, it is essential to detect and remove errors and inconsistencies due to an imperfect data collection process by introducing data cleaning techniques (Xiong et al., 2006) and data preprocessing approaches (García-Gil et al., 2019). At the same time, the expert knowledge base could be combined to label and classify the data to improve the quality and availability of the data (Alonso et al., 2012). Secondly, In the “quality control” phase, unified data standards and format specifications are established to ensure that data from different sources can be effectively integrated. Standards and formats that fit various devices and could be generalized and applied are currently urgent. Finally, In the “quality improvement” phase, data islands shall be prevented by strengthening agricultural digital infrastructure in a balanced manner—such as through public-funded expansion of rural broadband and IoT networks—and by promoting even distribution of data resources via regional agricultural data platforms that integrate and openly share key information like soil moisture, weather, and market data. Meanwhile, it is crucial to strengthen data collaboration among all stakeholders, including clarifying data ownership and rights, while ensuring data security and compliance during the sharing process.

In general, through the continuous improvement of the quality loop, the challenges such as data noise, data fog, and data islands faced by the application of AIGC technology in smart agriculture shall not be ignored, the efficiency and accuracy of data processing shall be emphasized and improved.
